# Robot-Aided Assessment of Wrist Proprioception

**DOI:** 10.3389/fnhum.2015.00198

**Published:** 2015-04-14

**Authors:** Leonardo Cappello, Naveen Elangovan, Sara Contu, Sanaz Khosravani, Jürgen Konczak, Lorenzo Masia

**Affiliations:** ^1^Department of Robotics Brain and Cognitive Sciences, Istituto Italiano di Tecnologia, Genova, Italy; ^2^School of Kinesiology, University of Minnesota, Minneapolis, MN, USA; ^3^School of Mechanical and Aerospace Engineering, Nanyang Technological University, Singapore, Singapore

**Keywords:** wrist proprioception, wrist robot, discrimination threshold, quantitative measurements, robotic rehabilitation

## Abstract

**Introduction:**

Impaired proprioception severely affects the control of gross and fine motor function. However, clinical assessment of proprioceptive deficits and its impact on motor function has been difficult to elucidate. Recent advances in haptic robotic interfaces designed for sensorimotor rehabilitation enabled the use of such devices for the assessment of proprioceptive function.

**Purpose:**

This study evaluated the feasibility of a wrist robot system to determine proprioceptive discrimination thresholds for two different DoFs of the wrist. Specifically, we sought to accomplish three aims: first, to establish data validity; second, to show that the system is sensitive to detect small differences in acuity; third, to establish test–retest reliability over repeated testing.

**Methodology:**

Eleven healthy adult subjects experienced two passive wrist movements and had to verbally indicate which movement had the larger amplitude. Based on a subject’s response data, a psychometric function was fitted and the wrist acuity threshold was established at the 75% correct response level. A subset of five subjects repeated the experimentation three times (T1, T2, and T3) to determine the test–retest reliability.

**Results:**

Mean threshold for wrist flexion was 2.15°± 0.43° and 1.52°± 0.36° for abduction. Encoder resolutions were 0.0075°(flexion–extension) and 0.0032°(abduction–adduction). Motor resolutions were 0.2°(flexion–extension) and 0.3°(abduction–adduction). Reliability coefficients were *r*_T2-T1_ = 0.986 and *r*_T3-T2_ = 0.971.

**Conclusion:**

We currently lack established norm data on the proprioceptive acuity of the wrist to establish direct validity. However, the magnitude of our reported thresholds is physiological, plausible, and well in line with available threshold data obtained at the elbow joint. Moreover, system has high resolution and is sensitive enough to detect small differences in acuity. Finally, the system produces reliable data over repeated testing.

## Introduction

Broadly defined, proprioception refers to the sense of body awareness. This awareness is based on signals from the receptors embedded in joints, muscles, tendons, and skin. Classically, four properties of proprioceptive function are distinguished: passive motion sense, active motion sense, limb position sense, and the sense of heaviness (Goldscheider, 1898).

It is well established that the processing of proprioceptive information is important for the neural control of movement. Conversely, the loss of proprioception negatively impacts the reflexive control of balance (Allum et al., 1998; Dietz, 2002; Rossignol et al., 2006) and severely impairs spatial (Gordon et al., 1995) as well as temporal aspects (Gentilucci et al., 1994) of voluntary movements. Numerous neurological and orthopedic conditions are associated with proprioceptive and kinesthetic impairments such as stroke (Langhorne et al., 2009; Coupar et al., 2012;), Parkinson’s disease (Rickards and Cody, 1997; Khudados et al., 1999; Mongeon et al., 2009), focal dystonia (Rosenkranz et al., 2000; Putzki et al., 2006), peripheral sensory neuropathies (Rothwell et al., 1982; Ghez et al., 1990), or injuries to ligaments, joint capsules, and muscles (Barrack et al., 1989; Lephart et al., 1994; Fridén et al., 1997).

Despite the recognition that proprioceptive deficits are the most frequent long-term side effects after stroke (Hunter and Crome, 2002), there is no established, precise method available in clinical settings to assess proprioceptive function. Clinical tests to assess proprioceptive acuity are coarse. For example, Nottingham Sensory Assessment (NSA) test (Lincoln et al., 1998) and Rivermead Assessment of Somatosensory Performance (RASP) test (Winward et al., 2002) are based on detecting a patient’s capability to discriminate the upwards or downwards position of a single limb segment (i.e., finger or toe) (Hagert, 2010). Although the loss of limb proprioception may severely impact the effectiveness of available rehabilitation protocols aiming to restore motor function (Kusoffsky et al., 1981; La Joie et al., 1982; Wade et al., 1983; De Weerdt et al., 1987; Stelzl et al., 1995), the presence of an objective, accurate, and reliable method to assess proprioceptive function is still missing in rehabilitation practice.

An alternative approach to assess proprioceptive function is to obtain psychophysical thresholds for joint position sense (JPS), motion sense (kinesthesia), and sense of tension or force. These threshold hunting methods yield two types of thresholds: a *detection threshold*, which is the smallest perceivable change in position, and a *discrimination threshold*, which is the just noticeable difference (JND) between two perceived positions (Gescheider et al., 1985). The detection threshold is considered a measure of the *sensitivity* while the discrimination threshold represents a measure of *acuity*. In contrast to joint matching methods that rely on active motion of the test person, threshold hunting paradigms often use specialized equipment that passively moves a person’s limb in a highly controlled manner (Deshpande et al., 2003; Maschke, 2003; Konczak et al., 2007; Westlake et al., 2007). Objective measurements of JPS have been obtained through the use of various instruments such as goniometers or inclinometers (Dover and Powers, 2003; Gay et al., 2010; Smith et al., 2013). Joint matching paradigms that mimic clinical testing have been most common to determine a JND threshold for JPS. However, recent research indicated that psychophysical threshold methods yield a more precise estimate of a limb position discrimination threshold than joint position matching methods, and passive motion testing results in lower thresholds than tests involving active motion (Elangovan et al., 2014).

Because threshold hunting requires the precise control of limb position and velocity, the use of haptic technology or robotic devices to passively move a limb has been introduced in recent years. For example, a planar robotic manipulandum was used to obtain motion sense thresholds of the hand of both healthy and stroke patients (Simo et al., 2011) and haptic acuity thresholds in children and patients with Parkinson’s disease (Gori et al., 2012; Konczak et al., 2012). A robotic sensory trainer evaluated JPS of the metacarpophalangeal joint (Lambercy et al., 2011). This system provided displacement, vibration, and pressure stimuli to the user’s finger for the assessment and therapy of hand sensory function. A bimanual robotic manipulandum was used to determine JPS thresholds in stroke subjects using a joint position matching paradigm where the robot passively placed one arm into a certain position and that the subjects used active movement to match this position with the other arm (Dukelow et al., 2010; Squeri et al., 2011).

Up to this point, most robotic devices have focused on testing single DoF joint such as the elbow, or were only capable of displacing or moving a joint in a single plane (i.e., dorsiflexion/plantarflexion of the ankle). This constraint restricts either the types of joints that can be investigated, or it provides only partial information on the proprioceptive status of a joint. However, clinically it is relevant to examine the proprioceptive function of all DoFs of a given joint or limb system. We, here, introduce the use of 3-DoF wrist robotic device to examine wrist joint proprioceptive acuity. Specifically, we applied a psychophysical threshold hunting method to determine JND thresholds for two different DoFs – flexion/extension and abduction/adduction (radial/ulnar deviation). The specific aims of the study were (a) to establish the validity of the system in producing threshold estimates that are in accordance to previously reported thresholds, (b) to demonstrate that the technology and implemented methodology are sensitive enough to produce distinct threshold values for each DoF, and (c) to establish test–retest reliability of the system.

## Materials and Methods

### Subjects

The experimental sessions were performed in two laboratories using the same instrumentation, at the School of Mechanical and Aerospace Engineering of Nanyang Technological University and School of Kinesiology of the University of Minnesota. Eleven right-handed young adults with no known neurological and neuromuscular disorders (mean age ± SD: 26.4 ± 3.4 years.) volunteered to participate the study. All participants gave their informed consent prior to testing. The experiments were conducted in accordance with the Universities ethical guidelines. The Edinburgh Handedness Questionnaire (Oldfield, 1971) was administered to determine handedness. All participants revealed a laterality index of >60 on a [-100 100] scale (mean ± SD: 82.7 ± 12.9), where -100 means completely left-handed and 100 completely right-handed, showing that they were right-hand dominant. Only the dominant right hand was evaluated.

### Apparatus

**Figure 1:**
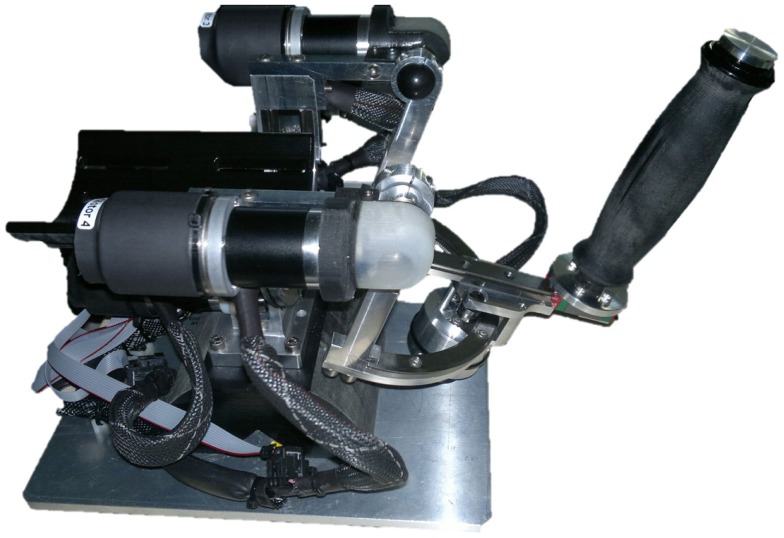
**The wrist robot: a three-DoF manipulandum used for the experiments**.

The Wrist Robot (Figure 1) is a three-DoF manipulandum (Masia et al., 2009). It allows full range of motion (ROM) for the human wrist. It is a fully backdrivable system that can deliver torque levels comparable to maximum isometric wrist torques of a human adult. The robot is powered by four brushless motors chosen in such a way to provide an accurate haptic rendering, and compensate for the weight and inertia of the device, or even overcoming muscular contraction and hypertonia. The continuous torque ranges at the different wrist joints are 1.53 Nm on FE, 1.63 Nm on AA, and 2.77 Nm on pronation/supination (PS). The RoM in the three DoFs approximately matches the RoM of a normal human wrist: 65°/70° of FE, 19°/30° of AA, 90°/90° of PS in a typical human subject vs. ±72° of FE, 45°/27° of AA; ±80° of PS in the wrist robot. Angular rotations on the three axes are acquired by means of 4000 quadrature-counts/revolution incremental encoders, resulting in a resolution of 0.0075 for FE DoF and 0.0032 for AA DoF. The system is integrated with a virtual reality environment (VR) providing the user with a visual feedback of his/her movement during the execution of haptic tasks. The control architecture is based on three control loops: (1) an inner loop running at 1 kHz in the motor servos; (2) an intermediate loop running at 1 kHz on a real time kernel that updates the current reference of each motor; (3) an external loop running at 100 Hz for the visual virtual reality and user interface. The device can thus deliver haptic position and velocity stimuli. The gain parameters of the PID controller running inside the motor drivers were tuned to deliver smooth movements necessary for the psychophysical threshold determination tests as described in the following section.

### Experiment

**Figure 2:**
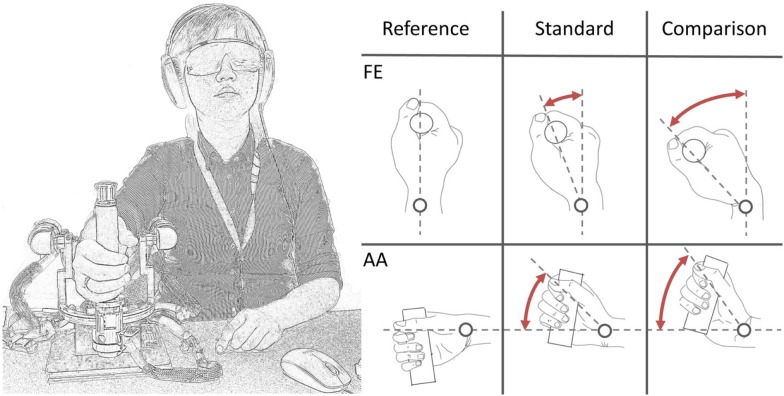
**A subject loosely grasped the handle of the wrist robot**. Auditory cues were masked by pink noise from headphones. Vision was occluded through opaque goggles. During each trial, a standard (=15°) and a comparison displacement (variable but >15°) was presented in random order.

Subjects sat next to the robot device (see Figure 2). The frontal plane of the body was aligned perpendicularly to the PS axis of the robot, which is horizontal. Seat position was adjusted in order to be comfortable for the participants, with the elbow angle of ~90°. Particular attention was given to the correct alignment of the wrist joint with the functional axis of the robot: to avoid joint misalignment and unwanted relative movements between the wrist and the robot during the experiment, subject’s forearm was firmly constrained to a rigid cast and secured by Velcro^®^ strips to the hardware support. Subjects were instructed to maintain a relaxed hand grip. Prior to testing the wrist, assumed a neutral joint position during FE condition; while in AA condition, the joint was adducted by 10° from neutral in order to prevent the device from reaching the anatomical limit of the workspace during stimulus presentation.

Vision was occluded by opaque glasses and hearing was masked by noise-canceling headphones to eliminate possible visual or acoustic cues. A unidirectional 2-alternative-forced-choice (2AFC) discrimination paradigm was chosen. Two different stimuli were presented in each trial: a 15° amplitude stimulus of fixed value (*standard* stimulus) and the other with variable amplitude across trials (*comparison* stimulus) and always higher than the standard (Figure 2). We will refer to *intensity* as the difference between the angular displacement of the two standard and comparison stimuli. The two stimuli were presented in random order, separated by a 2-second inter-stimulus interval. After each trial, the participant verbally indicated which stimulus was “larger” (i.e., which of the two movements had a larger displacement). Based on the subject’s response, a comparison stimulus was selected for the subsequent trial using an adaptive QUEST algorithm developed by Watson and Pelli (1983). In order to provide the subject with a more heterogeneous task, a random Gaussian noise was added for every trial to the comparison stimulus set to a maximum of ±20% of the current comparison stimulus itself. During each trial, the velocity of movement was kept constant at 6°/s.

### Measurements

The two conditions have been tested separately: each session lasted for ~45 min with 3 min rest after every 15–25 trials in order to prevent mental fatigue and enhance attention, a prerequisite for the validity of obtained psychophysical thresholds (Sullivan and Hedman, 2008). The intensity of the first trial was set to 7° in order to be easily detectable by all the subjects.

To obtain a proprioceptive threshold, the frequency of correct responses where the comparison stimulus was identified as larger than the standard stimulus was computed across the range of displayed stimuli. Response data were then fitted using a cumulative Gaussian function. The psychometric acuity function Ψ was computed for both the conditions, where Ψ describes the probability that a comparison at x is picked as the stimulus with the larger intensity. The psychometric function ranges from 50 to 100%. This implies that for low stimulus intensities the subject had a 50% of probability to give the correct answer, while for large intensities the comparison was correctly perceived as larger in 100% of trials (Wichmann and Hill, 2001). Based on Ψ, a discrimination threshold was defined as the intensity such that the subjects identified the comparison as larger with a frequency of 75% (McKee et al., 1985).

## Results

**Figure 3:**
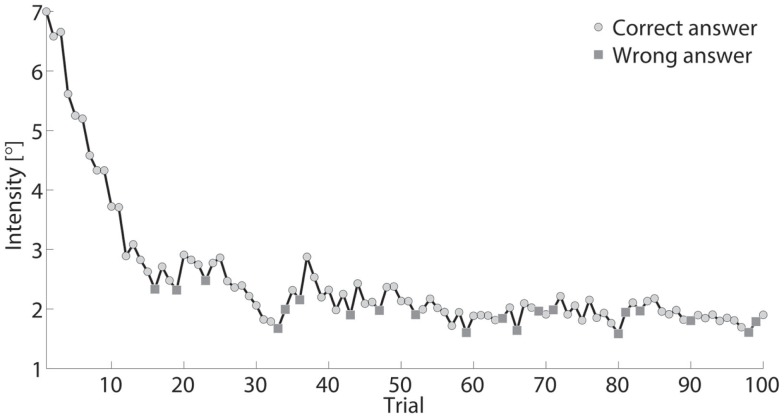
**Intensity, defined as stimulus difference size between comparison and standard stimuli, presented across 100 trials for one participant**. Standard stimulus was a constant (15°). Comparison stimulus rapidly decreased from 22° to ~17° during testing.

**Figure 4:**
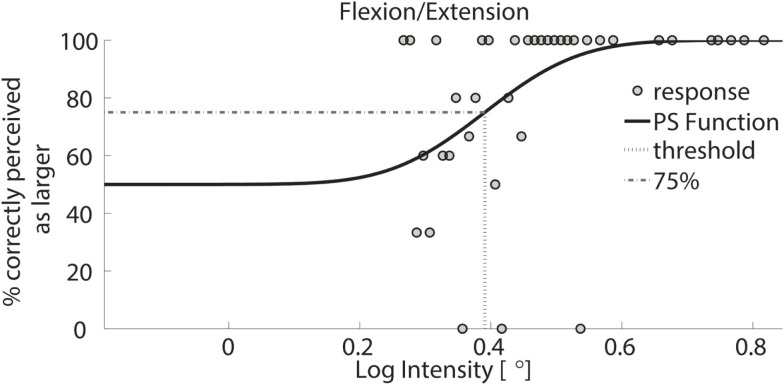
**Psychometric function of subject 1 for FE**. Circles represent binned responses recorded during the experimental procedure; vertical line depicts the mean (acuity threshold) of the Cumulative Gaussian. Data were discretized with a bin size of 0.01°; thus, every point can correspond to a variable amount of responses. The plot presents a threshold of 0.39 in logarithmic scale – corresponding to 2.46° in linear scale.

**Figure 5:**
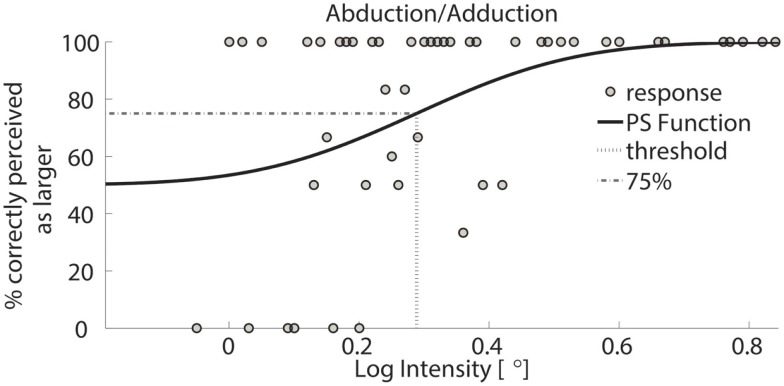
**Psychometric function of subject 1 for AA**. Circles represent binned responses recorded during the experimental procedure; vertical line depicts the mean (acuity threshold) of the Cumulative Gaussian. Data were discretized with a bin size of 0.01°; thus, every point can correspond to a variable amount of responses. The plot presents a threshold of 0.29 in logarithmic scale – corresponding to 1.94° in linear scale.

Exemplar response data of a single subject are shown in Figure 3. During testing, the differences between standard and comparison stimulus progressively converged toward a minimum, typically after approximately 40–70 trials. Data were visually inspected to verify the absence of lapsing errors in the upper asymptote, as these errors considerably affect the shape of the curve introducing bias in threshold estimates (Wichmann and Hill, 2001). Figures 4 and 5 show typical psychometric functions obtained for threshold detection in the two DOFs of the wrist joint.

**Figure 6:**
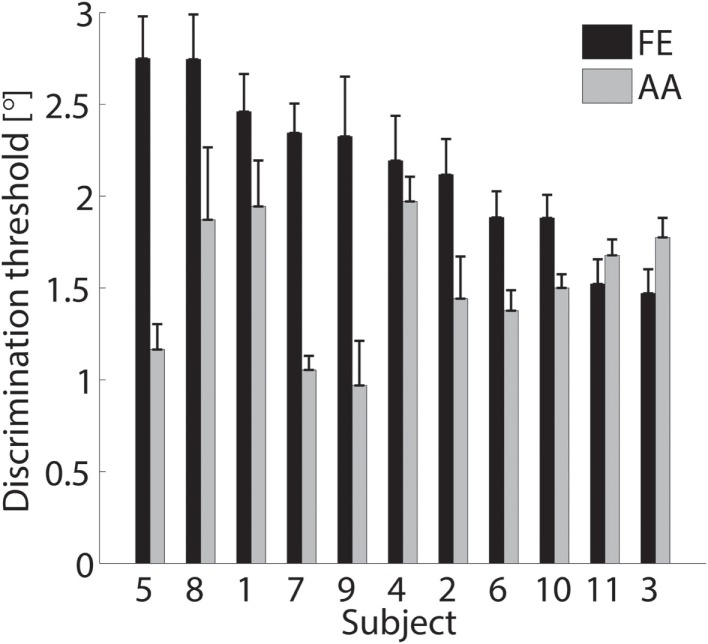
**Discrimination thresholds and the SDs of the sampled population**. Black bars refer to the FE condition while gray bars refer to AA. Data are sorted in descending order of FE threshold.

**Figure 7:**
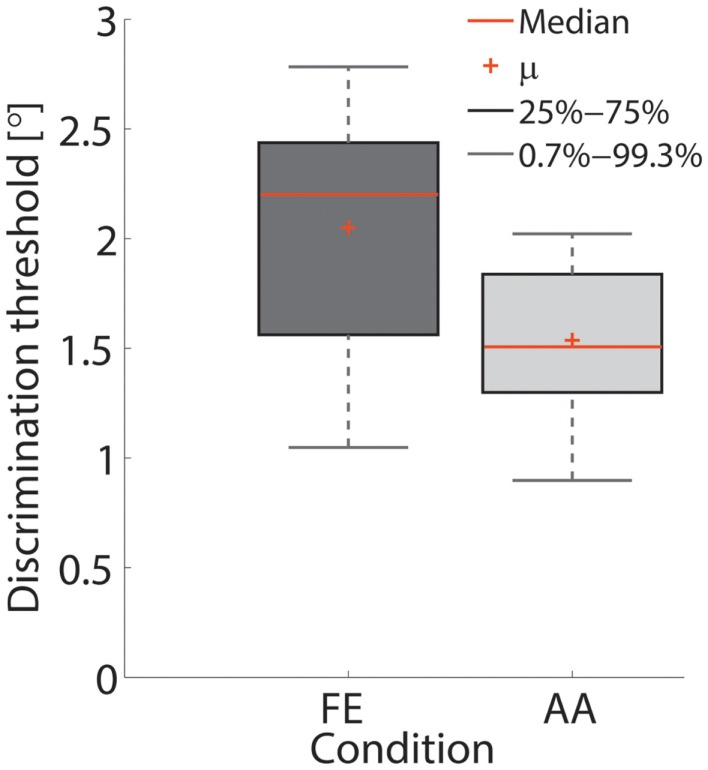
**Box-plot of the thresholds in the FE and AA conditions**. Red crosses depict the mean value across subjects.

**Figure 8:**
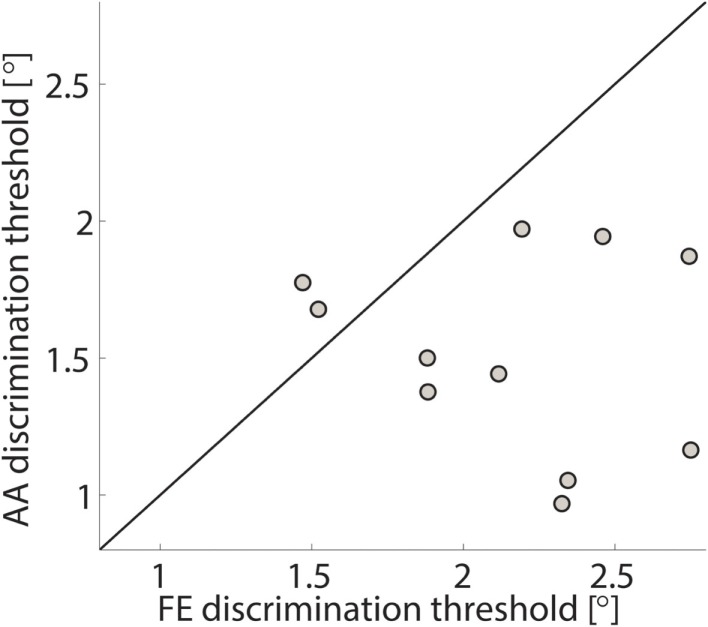
**FE discrimination thresholds vs. AA discrimination thresholds for the sampled population**. Each circle represents a subject and the line represents the bisector. The two subjects with a lower acuity in AA than in FE are ones with the highest acuity in FE.

The single subject data in Figures 4 and 5 reveal that this subject had a higher discrimination threshold for FE when compared to AA, yet was less certain about his judgments for AA (shallower slope of the function). With respect to the complete sample, nine of the 11 subjects exhibited FE thresholds that were higher than for AA indicating that both DoF have distinct acuities. Mean threshold for FE was 2.15° ± 0.43° and 1.52° ± 0.36° for AA. A subsequent one-way ANOVA indicated that the mean thresholds for each DF were significantly different from each other (*p* = 0.0013). Figures 6 and 7 summarize the thresholds for all the subjects, reporting the mean and the standard deviation. The two subjects with a lower proprioceptive acuity in FE compared to AA condition are the ones that performed best in the FE test: 1.47° and 1.52° for subjects 3 and 11, respectively, as better noticeable in Figure 8.

**Figure 9:**
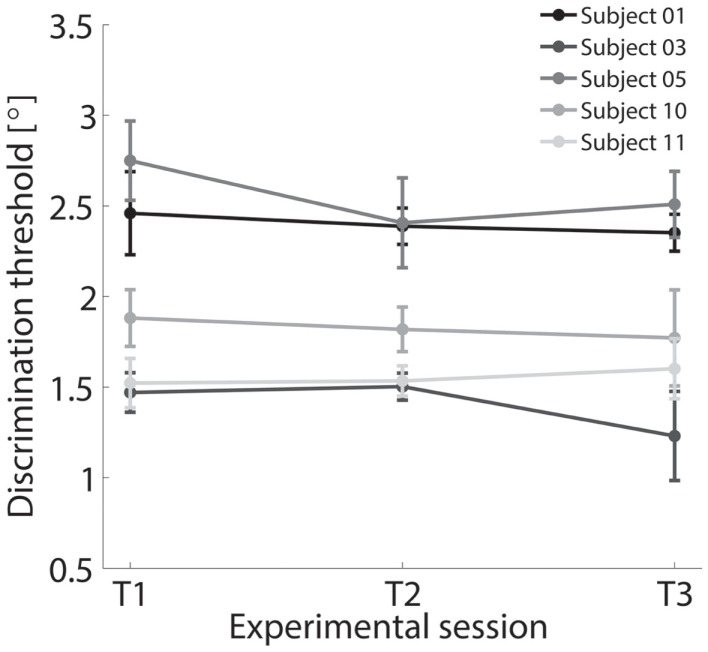
**Discrimination thresholds for FE condition across three different experimental sessions T1, T2, and T3 tested for a subset of five subjects**.

A subset of five subjects was retested under the identical experimental procedure for the FE condition resulting in a total of three different test sessions performed in three different days. Results are shown in Figure 9, where negligible inter-test variability is observable ensuring that the method is time independent and test–retest reliable.

## Discussion

We evaluated the feasibility of a three-degrees-of-freedom wrist robot system to determine proprioceptive discrimination thresholds for two different DoFs of the wrist. Specifically, we sought to accomplish three specific aims: first, to establish data validity meaning that the system produces measures of proprioceptive acuity that are in accordance to previously published results on proprioceptive acuity of the human wrist. Second, to show that the system is sensitive to detect small differences in acuity. Third, to establish values for the test–retest reliability of the system indicating that the approach provides reliable estimates of proprioceptive acuity over repeated testing.

### Mapping the proprioceptive acuity of the human wrist joint

Our approach yielded proprioceptive thresholds for two DoFs of the human wrist. Mean discrimination threshold for FE was 2.15° and 1.52° for AA. When one expresses these thresholds with respect to the standard displacement of 15°, the threshold for FE is about 14.3% of the size of the standard and approximately 10.1% for the AA DoF.

Proprioceptive acuity of the upper limb joints has been measured and reported by previous studies. Unfortunately, no norm data on human wrist joint acuity are available and most previous studies used a joint position matching paradigm to assess proprioceptive function. Employing such a joint position matching paradigm, Lephart et al. (1994) showed repositioning errors in the range of 12–31% of the target joint angle in normal shoulder joints. In a recent review, Goble (2010) reported absolute position matching errors in the magnitude of 2.5° for the elbow joint. Even though, these studies are not directly comparable with the current results because they reflect proprioceptive acuity of the elbow and not the wrist and were measured by joint position matching paradigms, they nevertheless serve as an estimate of the expected proprioceptive acuity of upper limb joints. A recent study by Elangovan et al. (2014) using the same psychophysical approach as our system reported a mean elbow joint discrimination threshold of 1.05°, which was approximately 10% of the standard of 10°. This finding coincides very closely with our results at the wrist joint. Moreover, given our approach uses a psychophysical method, our results should provide a more precise acuity measure for the wrist joint. The same study by Elangovan et al. (2014) also revealed that psychophysical thresholds were the most precise and least variable acuity measure. The psychophysical estimate was significantly lower than mean position errors obtained by ipsi- or contralateral joint position matching tasks (ipsilateral: 1.51°; contralateral: 1.84°) – a 44–75% difference in measurement accuracy. These findings underline that our measurements of wrist joint acuity are within the previously reported physiological range of upper limb acuity, demonstrating that the wrist robot system is capable of producing valid and accurate measures of wrist joint acuity.

Furthermore, we found that the acuity for AA is significantly higher than for FE. While this may be surprising on a first glance, it may be a very plausible finding if one considers the neuroanatomy of the human wrist joint. It is known that the ligaments stabilizing the wrist contain mechanoreceptors, Ruffini and Pacini-like corpuscles, which contribute to wrist proprioception. Immunohistochemical studies of the wrist joint ligaments revealed a rich distribution of mechanoreceptors in the dorso-radial ligaments such as dorsal radiocarpal, dorsal intercarpal, and scapholunate interosseous ligaments, a medium density in the volar and volar-triquetral ligaments, while others such as the long radiolunate ligament are nearly void of mechanoreceptors (Hagert et al., 2005, 2007). The highly innervated dorso-radial ligaments are stressed during AA, while the lesser innervated ligaments such as the volar ligaments get primarily stressed during FE. These differences in mechanoreceptor density and innervation may ultimately lead to differences in acuity, which is reflected in the differences in proprioceptive thresholds that we found. Furthermore, the AA DoF has a lower ROM than FE and forearm PS, and the presented stimuli (both standard and comparison) during the experiment scan a wide portion of the whole AA total range. The differences in the thresholds between the two DoFs highlighted the role of the structural differences in wrist joints, and application of robotic technology can unveil the anisotropy of proprioceptive acuity among the different human joint, providing more insights also in motor learning and explaining why particular pattern of muscular activations are preferred for determined tasks.

### Sensitivity of the system

Given that the thresholds are based on the verbal responses of a participant to a specific set of displacements, the sensitivity of the system is determined by the ability of the motors to create a precise displacement and by the sensitivity of the encoders recording the displacement. The resolution of the motors passively input the stimuli during the experiment is 0.2° for FE and 0.3° for AA, the resolution of the encoders is 0.0075° for FE and 0.0032° for AA. Motor and sensor resolution are well below the obtained proprioceptive thresholds, although it must be taken into account that quantization of motion might introduce a certain level of inaccuracy even if negligible. We cannot fully exclude that small misalignments of the wrist joint axes with the joint axes of the device affected the measurements. To assure consistency, at the beginning of each experimental session the subjects’ wrist was accurately positioned in order to properly match the axes of rotations. The low inter-test variability shown in Figure 9 underlines that the influence of anatomical-to-system joint misalignment was negligible.

Furthermore, the nature of the experimental paradigm (2-alternative-forced-choice discrimination paradigm) may also introduce a bias in participants if they are not correctly trained before initiating the test. We designed the experiment in order to present the two stimuli (standard and comparison) pseudorandom either the first or the second stimulus throughout the whole task. To evaluate subjects’ bias, we tested for the effect of stimulus order on the correct response, which showed no differences. Therefore, we can affirm that the inherent limitations of both robotic technology and experimental paradigm do not jeopardize the application of robotic technology in the proprioceptive assessment.

### Reliability of the system

To assess test–retest reliability of the threshold estimates, we repeated the procedure for two additional times (T2 and T3) in five subjects only for the FE condition. The coefficients of test–retest reliability were *r* = 0.986 for T2 with respect T1 and *r* = 0.971 for T3 with respect to T2. The mean within-subject variability across all three tests was *s* = 0.09°. The results highlight excellent reliability. The proposed approach thus is repeatable, a key attribute of a quantitative measuring system. Given the precision of the motors and sensors, the most import source of variability of the threshold estimates across the sessions is likely the variability of a subject’s verbal responses across different test dates to identical stimuli.

### Usefulness of the wrist robot for diagnostics

Although, robotic technology has been widely promoted for use in rehabilitation (Prange et al., 2006; Dechaumont-Palacin et al., 2008), its application for diagnostics of proprioceptive function is still in its infancy. To our knowledge, no previous studies using haptic-capable robotic devices reported wrist proprioceptive discrimination thresholds for JPS. Based on established psychophysical assessment methods known to produce reliable and accurate results for quantification of proprioceptive discrimination thresholds (Elangovan et al., 2014), we employed a robotic device to accurately deliver in a repetitive way position stimuli in two different anatomical planes of movement and consequently measured wrist proprioceptive acuity. Our findings provide evidence for the feasibility of robotic-aided proprioceptive assessment.

### Multi-platform portability and future work

Our data demonstrate that our robotic manipulandum is well suited to perform the proprioceptive assessment. Nevertheless, the same approach can be implemented in different physical platforms should their performances be comparable with the ones of our device. Mandatory characteristics of other robots to perform the proposed assessment hence are: high encoder resolution, elevate positioning accuracy, and wide torque range. Therefore, the robotic manipulanda presented by Krebs et al. (2004) and Pehlivan et al. (2014) are good examples of platforms able to replicate the proposed method. Such a portability expands the potentiality of the assessment, which could transcend the particular hardware and become a standard method in the (robotic) rehabilitation practice allowing the physiotherapists to collect standardized data and compare them with many others collected worldwide.

To compare data, however, a full characterization of wrist proprioceptive acuity is necessary. For this reason, investigations on wrist PS as well as wrist abduction and extension shall be performed in the future as next research steps. Similarly, the influence of the initial position and of the value of the standard stimulus shall also be investigated. Further studies can take in account the evaluation of proprioceptive functions of the non-dominant hand, as well as of multi-DOF motions. A database describing the proprioceptive functions of intact wrist joint could be thus gradually made.

Furthermore, we propose a standard paradigm for proprioceptive discrimination thresholds in human upper limb which should not be limited to the distal part of the arm but it should be extended to different anatomical districts for multi-joint investigation in future studies.

## Conclusion

The data collected by the Wrist Robot allowed us to determine the proprioceptive acuity thresholds of FE and AA DoFs of human wrist joint in healthy subjects. Beyond its neuroscientific relevance, this result introduces, for the first time, the use of a robotic interface to assess proprioceptive acuity of the wrist joint. We showed that the technology can generate robust, reliable, and unbiased measures of proprioceptive function that allow for the efficient quantification of proprioceptive status and dysfunction. The use of a robotic system provides multiple advantages: it increases measurement resolution and precision, has good test–retest repeatability, avoids the problem of poor inter-rater reliability common in many clinical scales, and reduces the variability of the reported outcome measures. Consequently, if properly employed, it can reduce costs by reducing the reliance of a clinician or therapist to obtain proprioceptive diagnostics.

Our findings support the use of an integrated robotic device to deliver rehabilitation in the form of sensory and motor intervention as well as to assess the progress in proprioception occurring as a result of the robotic intervention in an unbiased manner. Such a device apart from providing multimodal sensory feedback (visual, tactile, and haptic) can also be used to deliver and modify treatment interventions based on the monitored progress in proprioceptive recovery. An integrated haptic robotic device that can assess proprioception, monitor patient progress in proprioception, and deliver rehabilitation training may increase the efficiency of training and reduce the amount of individual attention needed from the clinician. This integration can be implemented with additional software and minimal hardware enhancements. Although, this integrated device will automate the assessment and rehabilitative procedure, it may not entirely replace a clinician who can deliver sophisticated personal human interaction.

## Conflict of Interest Statement

The authors declare that the research was conducted in the absence of any commercial or financial relationships that could be construed as a potential conflict of interest.
